# Identification of genome-wide non-canonical spliced regions and analysis of biological functions for spliced sequences using Read-Split-Fly

**DOI:** 10.1186/s12859-017-1801-y

**Published:** 2017-10-03

**Authors:** Yongsheng Bai, Jeff Kinne, Lizhong Ding, Ethan C. Rath, Aaron Cox, Siva Dharman Naidu

**Affiliations:** 1 Department of Biology, Indiana State University, 600 Chestnut Street, Terre Haute, IN 47809 USA; 2 The Center for Genomic Advocacy, Indiana State University, 600 Chestnut Street, Terre Haute, IN 47809 USA; 3 Department of Mathematics and Computer Science, Indiana State University, 200 North Seventh Street, Terre Haute, IN 47809 USA

**Keywords:** Read-Split-Fly, Alternative splicing, Non-canonical, RNA-Seq, ENCODE

## Abstract

**Background:**

It is generally thought that most canonical or non-canonical splicing events involving U2- and U12 spliceosomes occur within nuclear pre-mRNAs. However, the question of whether at least some U12-type splicing occurs in the cytoplasm is still unclear. In recent years next-generation sequencing technologies have revolutionized the field. The “Read-Split-Walk” (RSW) and “Read-Split-Run” (RSR) methods were developed to identify genome-wide non-canonical spliced regions including special events occurring in cytoplasm. As the significant amount of genome/transcriptome data such as, Encyclopedia of DNA Elements (ENCODE) project, have been generated, we have advanced a newer more memory-efficient version of the algorithm, “Read-Split-Fly” (RSF), which can detect non-canonical spliced regions with higher sensitivity and improved speed. The RSF algorithm also outputs the spliced sequences for further downstream biological function analysis.

**Results:**

We used open access ENCODE project RNA-Seq data to search spliced intron sequences against the U12-type spliced intron sequence database to examine whether some events could occur as potential signatures of U12-type splicing. The check was performed by searching spliced sequences against 5’ss and 3’ss sequences from the well-known orthologous U12-type spliceosomal intron database U12DB. Preliminary results of searching 70 ENCODE samples indicated that the presence of 5’ss with U12-type signature is more frequent than U2-type and prevalent in non-canonical junctions reported by RSF. The selected spliced sequences have also been further studied using miRBase to elucidate their functionality. Preliminary results from 70 samples of ENCODE datasets show that several miRNAs are prevalent in studied ENCODE samples. Two of these are associated with many diseases as suggested in the literature. Specifically, *hsa-miR-1273* and *hsa-miR-548* are associated with many diseases and cancers.

**Conclusions:**

Our RSF pipeline is able to detect many possible junctions (especially those with a high RPKM) with very high overall accuracy and relative high accuracy for novel junctions. We have incorporated useful parameter features into the pipeline such as, handling variable-length read data, and searching spliced sequences for splicing signatures and miRNA events. We suggest RSF, a tool for identifying novel splicing events, is applicable to study a range of diseases across biological systems under different experimental conditions.

**Electronic supplementary material:**

The online version of this article (10.1186/s12859-017-1801-y) contains supplementary material, which is available to authorized users.

## Background

Alternative splicing (AS) is an important posttranscriptional process enabling a single gene to generate multiple different transcripts, also called isoforms [1, 2]. AS can increase the proteome diversity as well as modulate the stability of mRNAs by means of downstream RNA quality control (QC) mechanisms, which include nonsense-mediated decay (NMD) of the transcripts that possess premature termination codons and nuclear retention and elimination (NRE) of transcripts that contain introns [3]. In eukaryotes, the AS process removes introns from the nuclear pre-mRNAs with the help of the spliceosome, which can recognize conserved short consensus sequences within the introns and at intron-exon boundaries. More specifically, conserved dinucleotides located at the first two and the last two positions of introns in the pre-mRNAs are recognized by the spliceosome [4].

In higher eukaryotes, there are two types of identified spliceosome complex that catalyze the pre-mRNA splicing [5]. The majority of pre-mRNA introns (U2-type introns) are excised by the U2-dependent, major spliceosome that is found in all eukaryotes, whereas approximately 0.35% of human introns (U12-type introns) were removed by the U12-dependent, minor spliceosome that is found in only a subset of organisms [6–8]. Approximately 700 to 800 genes containing U12-type introns were identified in the human genome [9]. Unlike U2-type introns, the U12-type introns lack a polypyrimidine tract that is located upstream of the 3′ splice site (ss) however, the U12-type introns have highly conserved sequences located at their 5′ ss as well as branch sites [6]. It was found that, within the same gene, the U12-type introns co-occur with the U2-type introns, but the U12-type introns are spliced more slowly, suggesting the role of U12-type splicing in a rate-limiting step in gene expression [10]. The U12-dependent spliceosome is composed of the U11, U12, U4atac, and U6atac snRNPs, which are the functional homologs of the U1, U2, U4, and U6 in the U2-dependent spliceosome, respectively. Both U2-type and U12-type spliceosomes have the U5 snRNP [5, 8]. Although U2-type and U12-type spliceosomes have most of their protein components shared, seven protein components are unique and associated with the U11/U12 di-snRNP so that the U11/U12 di-snRNP can recognize the branch point sequences and the 5′ splice sites of the U12-type introns [8].

Mutations in the U12-type spliceosome, either in specific snRNA or in protein components, can cause diseases of very narrow tissue-specific consequences [11, 12]. Three patients possess severe isolated growth hormone deficiency (IGHD) and pituitary hypoplasia that arise from the biallelic mutations in the RNPC3 gene that encodes the 65 kDa protein component of the U12-type spliceosome [12]. Mutations in specific regions in the U4atac snRNA cause microcephalic osteodysplastic primordial dwarfism type I (MOPD I), also called Taybi-Linder syndrome (TALS). The mutations most probably result in distortion of the phylogenetically conserved stem-loop (SL) structure formed by U4atac snRNA. The distortion prevents the normal binding of a 15.5 K protein component of the spliceosome to the SL structure, thereby causing a series of downstream consequences, and eventually accumulating the immature pre-mRNAs that carry unspliced U12-type introns [13].

MicroRNA (miRNA) are small, non-coding RNA that serve as genetic regulatory elements in animals by silencing, and in rare cases enhancing, other mRNA transcripts. These single-stranded RNA are approximately 22 nucleotides in length and are involved in many processes throughout the body [14, 15]. These small mature miRNA are processed from longer pre-miRNA. Pre-miRNA forms a stem and loop structure that is processed by two RNase III enzymes: Drosha and Dicer [16]. Regulation mediated by miRNA targets mature mRNA in the cytoplasm, the miRNA will bind to the 3’UTR of the target mRNA. This binding can help to stabilize the targeted transcript but is usually followed by the interaction with RISC complex which leads to degradation of the mRNA, by this process miRNA is able to effectively silence the translation of its target [17]. Beyond degradation, miRNA also physically impairs the binding of the mRNA to the ribosome [14, 15]. It is estimated that more than 60% of all protein coding genes are regulated by miRNAs by these methods [18]. As such, miRNA has been implicated in many different complex diseases.

These diseases include many cancers, neurological diseases, cardiovascular disease, and other inheritable diseases. In cancer miRNA expression profiles have been well documented with many differences in expression between normal and tumor tissue. Typically this is shown by an overall downregulation of miRNA in tumor tissues [19]. miRNAs are able to act as either tumor suppressor genes or oncogenes depending on the targets of the specific miRNA [20–23]. The development of neurons is highly influenced by the presence of miRNA [24]. As such, the misregulation of miRNA has been implicated in Parkinson’s disease, Alzheimer’s disease, Down’s syndrome, and many other diseases [25–33]. The involvement of miRNA in cardiovascular diseases is similar to their involvement in neurological diseases - changes in miRNA expression can lead to arrhythmias, vascular abnormalities, unrestricted muscular growth, hypertension, and can lead to death if completely removed [34–42]. Other diseases that have been associated with miRNA include 5q syndrome, ICF syndrome, Rett’s syndrome, Crohn’s disease, and even deafness [43–48]. With the implications of miRNA in a multitude of complex diseases they have become important for targeted therapies and potential indicators of these diseases making them an important target for further study.

Given that such types of non-canonical splicing events of short mRNA regions and U12-type intron are important across biological systems and diseases, there is an urgent need to develop methodologies for identifying all possible non-canonical short splicing regions in cytoplasm and also looking for U12-type spliced isoforms. Most existing tools for detecting next-generation sequencing-based splicing events focus on generic splicing events. Consequently, non-canonical splicing events of short mRNA regions occurring within the cytosol and U12-type events have not yet been thoroughly investigated using bioinformatics approaches in conjunction with next-generation technologies at a genome-wide level.

We have developed a novel bioinformatics pipeline method named the Read-Split-Walk (RSW) [49] and Read-Split-Run (RSR) [50] for detecting non-canonical, short, splicing regions using RNA-Seq data. In this study, we have advanced the algorithm with an improved running speed and memory usage. We have applied RSF on human ENCODE data to characterize U12 splicing and study miRNA signatures in spliced sequences.

## Results

### RSF pipeline

**Fig. 1:**
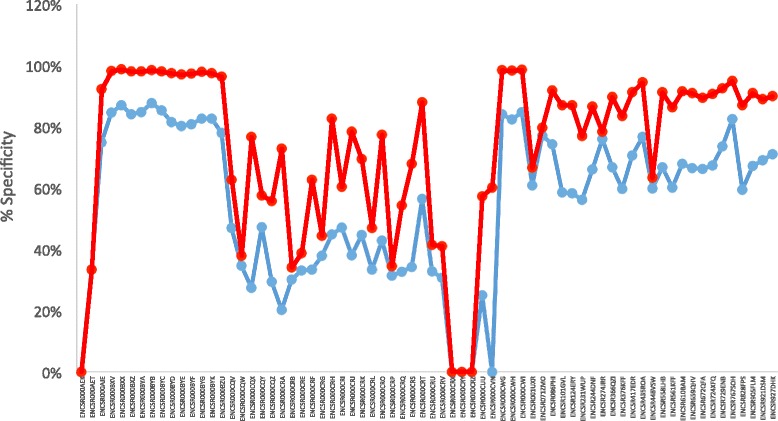
Specificity measured for all detected junctions (*red*) and for just novel junctions (*blue*) across 70 ENCODE samples

**Fig. 2:**
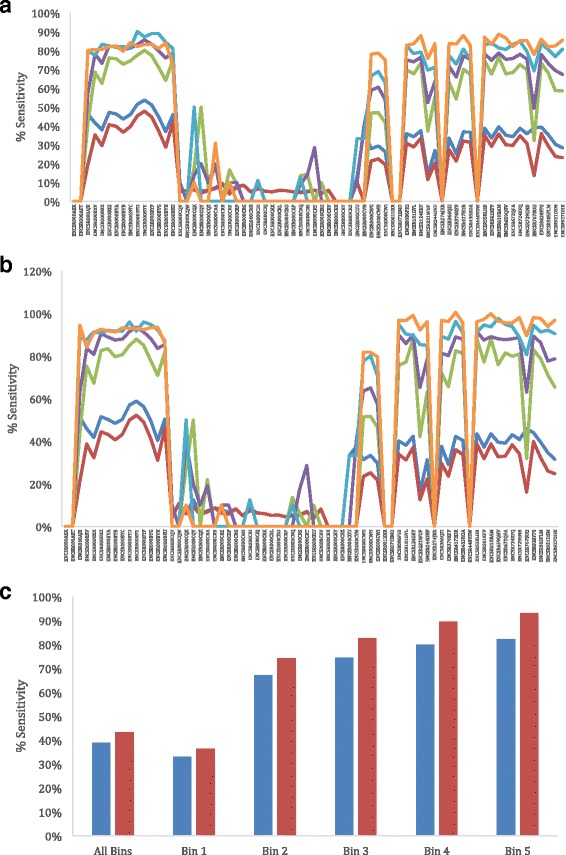
Measure of sensitivity of RSF across 70 samples. (**a**) Sensitivity measured for all known junction across 70 different samples separated by RPKM of supporting reads. All detected and possible junctions (*blue*), Bin 1 (*red*) RPKM <5, Bin 2 (*green*) RPKM 5–10, Bin 3 (*purple*) RPKM 10–50, Bin 4 (*light blue*) RPKM 50–100, and Bin 5 (*orange*) RPKM >100. (**b**) Sensitivity for the genes detected by RSF with a single isoform, bins same as above. (**c**) Total sensitivity of all genes across all samples in each bin (explained above) for all genes detected (*blue*) and only single isoform genes detected (*red*)

The presence of novel isoforms by splicing independent of normal mRNA processing has previously been identified by the Read-Split-Walk (RSW) pipeline developed in 2014 [49] and Read-Split-Run (RSR) [50]. Here we developed an updated version of RSF: Read-Split-Fly. This enhanced RSF has a newly developed pipeline with improved performance, sensitivity, and flexible parameter features. This pipeline has achieved a reasonable specificity (>60%) of novel junctions for the half of tested ENCODE samples and high specificity for detection of both known and novel junctions for ¾ of tested ENCODE samples, with some samples having as high as 98% specificity (Fig. 1). The lower bound of the sensitivity of RSF was calculated using the UCSC refFlat file, this resulted in a total of sensitivity across all samples tested (39%) with a maximum sensitivity of 90% (Fig. 2a). Calculating the sensitivity only for genes with a single isoform and detected by RSF resulted in a slightly higher sensitivity calculation overall (Fig. 2b). An analysis was also performed for genes grouped by RPKM. As expected, RSF has a much higher sensitivity in detecting high RPKM genes than those with a low RPKM (Fig. 2a-c).

### Comparison of detected spliced regions between RSR and RSF

**Fig. 3:**
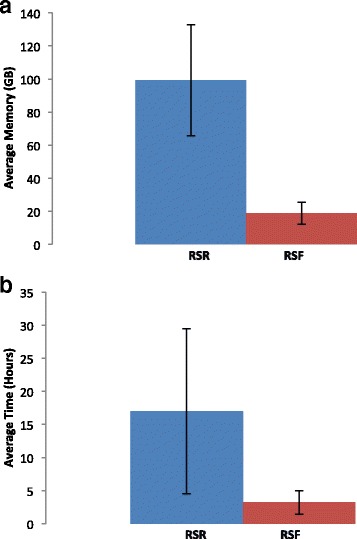
Comparison of average memory usage (*a*) and average running time (**b**) between RSR (*blue*) and RSF (*red*) for 66 ENCODE samples. *Error bars* show a single standard deviation

RSR and RSF were both run on the same 70 samples from the ENCODE dataset in order to compare their performance and sensitivity. The efficiency of RSF over RSR is evident in the amount of memory and CPU time that each sample requires to complete its run. RSF showed an average of four-fold decrease in needed CPU time and a threefold decrease in the required memory (Fig. 3 and Additional file 1). Along with improvements in the efficiency of the program, RSF is able to detect more spliced regions than RSR. RSF can detect 6% more spliced regions than RSR reports as well as more unique junctions (Table 1).

**Table 1:** Comparison of junctions detected by RSR and RSF

	RSR	RSF
Entries	146,769	155,021
Unique Splices	15,762	24,014
Splice junctions in common	131,007	

### The spliced regions detected by the RSF pipeline for human ENCODE data

The RSF pipeline was used to identify spliced regions that exhibit different signature in cancer versus normal samples from the ENCODE dataset. The analysis was performed using 28 cancer samples and 42 normal samples from ENCODE (Additional file 1). Two hundred ninety-seven spliced regions were found to occur in a higher percentage (greater than 50% difference) of cancer samples than normal samples; of these, 26 were detected within at least 55% of the cancer tissue samples, with the greatest occurrence being 86%. These were further classified by their specific tissue type. Specifically, all of them were found in samples associated with the adenocarcinoma and breast cancer, and some subset of the same 297 junctions were found in the other types of cancer (Table 2). Six hundred eleven unique splice junctions were found that occurred in a much higher percentage (greater than 50% difference) of normal samples than cancer samples; of these, 168 were detected within at least 55% of the normal tissue samples (Additional file 2). These results are based on the normalized comparison. These shared splice junctions show great potential for further analysis of their importance in the development of these specific cancers and in general tumor formation.

**Table 2:** Number of unique splice junctions with at least 50% greater frequency in Cancer samples than Normal samples, and vice versa

Total unique junctions (Cancer)	297
Adenocarcinoma	297
Neuroblastoma	1
Cervical Cancer	2
Breast Cancer	297
Leukemia	294
Total unique junctions (Normal)	611

### The downstream analysis for spliced sequences of RSF algorithm

Using various splicing categories of downloaded U12 and U2-type intron queries against the junctions as queries, we blasted the queries against the splice junction sequences reported by RSF from 70 ENCODE samples. We found that U2-type intron hits are much more than the U12-type hits, consistent with the major proportion of U2-type introns and minor proportion of the U12-type introns. The 5p_full queries got less hits than the 3p_full queries in both U12 and U2 type introns. Interestingly, both U12-type and U2-type intron 5p_full queries hits more novel splicing junctions relative to the known splice junctions, whereas both U12-type and U2-type 3p_full queries hits less novel splicing junctions relative to the known splice junctions. We also observed that there are more U12-type than U2-type for 5p_full category (Table 3). We didn’t have branch queries for U2, which were not listed in the U12DB website [51].

**Table 3:** Number of junctions reported by RSF for various splicing categories of U12 and U2-type

	U12			U2			Grand Total
Query	Known Sequences	Novel Sequences	U12 Total	Known Sequences	Novel Sequences	U2 Total	
3p_Full	6583	742	7325	823	155	978	8303
5p_Full	204	272	476	14	47	61	537
branch	2357	386	2743	0	0	0	2743
Grand Total	9144	1400	10,544	837	202	1039	11,583

**Fig. 4:**
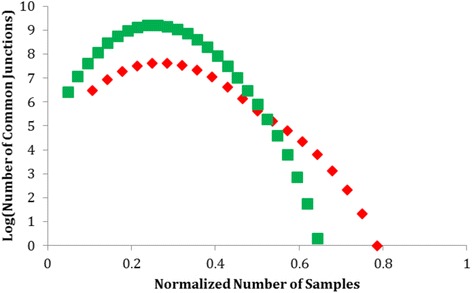
The miRBase miRNA hits for 70 ENCODE samples (Only miRNAs that have > = 30 hits are labeled)

For the spliced junctions found by RSF in 70 human ENCODE samples, the spliced sequences were exported for further analysis. *Homo sapiens* miRNA sequences were downloaded, and a custom shell script was written to run BLAST to report the number of hits of each miRNA sequence within each ENCODE spliced sequence. The total number of hits of each miRNA sequence over spliced sequences from all 70 ENCODE samples is reported in Fig. 4 and Additional file 3. Two hundred twenty-one miRNAs have at least 1 hit among the spliced sequences from 70 ENCODE samples. Seven miRNAs hits were seen within the spliced sequences of at least 30 out of 70 ENCODE samples. Of these seven, *hsa-miR-1273d*, *hsa-miR-548aa*, *hsa-miR-548 t-3p*, and *hsa-miR-1273 g-3p* have known associations with different cancer types. The cancer association results are summarized in Table 4.

**Table 4:** Top hits for disease associated miRNAs

miRNA name	Hits	Associated diseases
*hsa-miR-1273d*	64	Disease Progression Lymphoma, Large B-Cell, Diffuse 0Melanoma Neoplasm Metastasis Neoplasms Skin Neoplasms Uterine Cervical Neoplasms
*hsa-miR-548a*	46	Acute Disease Carcinoma, Hepatocellular Cell Transformation, Neoplastic Chromosome Deletion Colorectal Neoplasms Cri-du-Chat Syndrome Disease Progression Glioblastoma Hematologic Neoplasms Liver Neoplasms Lymphoma, Large B-Cell, Diffuse Melanoma Microsatellite Instability Multiple Sclerosis Neoplasm Metastasis Neoplasms Neoplasms, Glandular and Epithelial Ovarian Neoplasms Prostatic Neoplasms Pulmonary Embolism Skin Neoplasms
*hsa-miR-548 t-3p*	46	Acute Disease Carcinoma, Hepatocellular Cell Transformation, Neoplastic Chromosome Deletion Colorectal Neoplasms Cri-du-Chat Syndrome Disease Progression Glioblastoma Hematologic Neoplasms Liver Neoplasms Lymphoma, Large B-Cell, Diffuse Melanoma Microsatellite Instability Multiple Sclerosis Neoplasm Metastasis Neoplasms Neoplasms, Glandular and Epithelial Ovarian Neoplasms Prostatic Neoplasms Pulmonary Embolism Skin Neoplasms
*hsa-miR-619-5p*	31	Not Available
*hsa-miR-1273 g-3p*	30	Disease Progression Lymphoma, Large B-Cell, Diffuse Melanoma Neoplasm Metastasis Neoplasms Skin Neoplasms Uterine Cervical Neoplasms
*hsa-miR-5096*	30	Not Available
*hsa-miR-5585-3p*	30	Not Available

## Discussion

### Parameter consideration in RSF pipeline

Several parameters allow the user to customize RSF output, but have little effect on the time or resources needed to execute the pipeline. These include MODE and SUPP. MODE must be “analytic” or “comparison”. Single datasets are run in analytic mode. Comparison provides a side-by-side comparison of common and unique splice junctions in addition to the standard output for each dataset. Any potential splice junction site must carry a minimum of SUPP supporting reads to be reported.

The MIN_D and MAX_D parameters control the minimum and maximum distance for which split reads are reported by RSF. In general, larger values may increase both sensitivity and the running time of the pipeline. MAX_ALIGNMENTS determines the number of alignments before a read or partial read is ignored due to having too many alignments. A higher value may increase sensitivity and running time. MIN_SPLIT_SIZE determines the smallest length that a read is split into for mapping to the reference genome. A lower value increases sensitivity and running time. Running time can be made quite small for test runs by setting both MAX_D and MAX_ALIGNMENTS very low and MIN_SPLIT_SIZE very high (e.g., 10,000, 2, and just under half of the original read length, respectively).

### Disk utilization of our RSF algorithm

For each RNA-Seq read that originally fails to align to the reference genome, there is a quadratic increase in storage requirements which scales relative to the minimum split size selected. With a minimum split size of 11 nt selected, a relatively small FASTQ file containing 7.8 million unmapped reads measuring 50 nt each would become a file with 453.4 million reads measuring between 11 and 39 nt. In terms of disk space usage, this particular file expands from 1.2 to 53.2GB. Since these files can rapidly fill up even large hard drives, it is important to delete files as they become unnecessary. Our pipeline has been developed to automatically delete alignment output files generated from two steps of bowtie. This use of inexpensive disk space to handle intermediate data lends itself to a more memory-efficient program.

### RSF running speed, sensitivity, and specificity

With these improvements RSF produces results for larger datasets in much less time allowing for more data to be processed and a better and more thorough understanding of potential novel splice junctions. RSF also can process RNA-Seq files containing variable-length reads, which makes our software be more flexible in handling data generated from Ion Torrent sequencer. The low sensitivity of certain files was directly correlated with the number of junctions that were reported by RSF as well as the RPKM. Genes with a low RPKM had much lower sensitivity (31%), while those with a high RPKM showed a much higher total sensitivity (82%) (Fig. 2c). Sensitivity calculations can be artificially low for genes with many isoforms that are not expressed. For genes with only a single isoform in the UCSC ref.-flat file, the sensitivity of RSF ranged from 39% for low RPKM genes to 93% for genes with high RPKM (Fig. 2b-c). Conversely, the specificity for RSF to accurately detect all junctions and novel junctions are relatively high. Overall, RSF is able to detect many possible junctions (especially those with a high RPKM) with very high overall accuracy and rather high accuracy for novel junctions.

### Applying RSF on human ENCODE RNA-Seq data

**Fig. 5:**
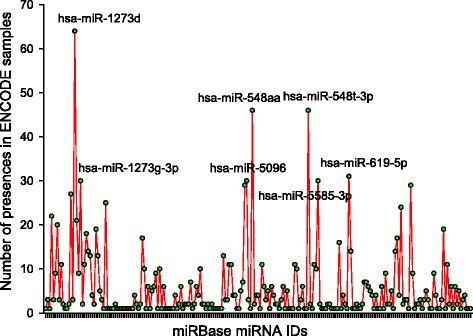
A display of the number of common junctions for cancer (*red diamond*) and normal samples (*green squares*) that are present in a given percentage of samples for each class (cancer and normal). Number of samples in each category is normalized by dividing the number of samples by the total amount of samples in that class (*Green*: Normal; *Red*: Tumor)

The splicing events identified by RSF on the 70 human ENCODE samples used for this project, yielded many potential avenues for further research. The novel splice junctions (Additional file 2) are especially of great interest. These splicing events were present in cancerous tissues making the transcripts and their potential protein products good candidates for the study of cancer development. Because of their novel nature, the impact of these splicing events cannot be ascertained at this moment, but we are hopeful in the impact of the discovery of these and many more using RSF. The previously known splicing events are also an interesting avenue of research as they are still expressed differently between cancerous and normal tissue. It is also interesting to note the distribution of shared splicing events, the normal tissue has the most shared splicing events around a quarter of all samples and quickly tapers off from there (Fig. 5). The cancerous samples however, have two notable maxima, one at 0.3 and another at 0.55, making the curve taper at a much steadier rate. From this we can reasonably conclude that the cancerous tissue seems to have a greater number of shared sequences for a higher percentage of its samples (Fig. 5).

## Conclusions

We have developed an improved RSF pipeline that can detect novel splicing events with better performance and accuracy when compared to previous RSW and RSR methods. Our RSF allows flexible parameters and can process large number of samples in a memory efficient manner.

## Methods

### The reference genome for Read-Split-Fly

The RSF program uses the *Homo sapiens* GRCh37/hg19 genome as its reference for all bowtie related alignment and splice junction detection. This genome is freely available for download from the University of California Santa Cruz Genome browser [52].

### Read-Split-Fly Algorithm

RSF shares the same basic framework that was developed in earlier work [49, 50]. In brief, (i) short reads of RNA-Seq data are mapped to a reference genome with the bowtie aligner, (ii) unmapped reads are split at various points, (iii) the read parts are mapped to the reference genome, (iv) mapped parts that are from the same original read and map within the same gene are called a matched pair, and (v) all matched pairs are compared to determine which support each other and which splice junctions have a high amount of support. See [50] for more details on the basic framework.

RSF has a number of key improvements over the earlier RSR and RSW pipelines – allowing files with variable length reads, improved speed and memory efficiency, increased sensitivity, and incorporation of downstream analysis. The entire pipeline is updated so input files can contain reads of variable length; this is done by modifying each part of the code to take the read length into account (previously the read length was a parameter fixed during the processing of a given input file). The calculation of supporting reads is performed in a faster and more memory-efficient manner. Speed and memory usage are improved by sorting candidate splices by both ends of the splice and processing all matched pairs in a given region of the genome at once.

Sensitivity is improved in the pipeline by taking into account sub-sequences of unmapped reads that do not result in any matched pair. Unmapped reads are split into two parts (a left and right side) and aligned to the reference genome. In some circumstances, one part aligns to many locations; for example this occurs if one side is very short (e.g., if the read is split into left side of length 8 and right side the remaining nucleotides). In these situations, both RSR and RSF ignore the part that aligns to many locations for reasons of efficiency. RSF rescues these reads by including the remaining longer side of the read when computing supporting reads of matched pairs – alignments of the long side of reads that have no matched pairs are compared against candidate splices, and are counted as supporting a matched pair if the longer side aligns with one end of the splice.

The RSF pipeline includes the option of including downstream analysis by searching databases of U12 and U2-type splice sequences and miRNA sequences within the splices found by the pipeline, as described further below.

### Methods of running Read-Split-Fly on human ENCODE dataset

Using RSF over 150,000 unique splice junctions were detected within the ENCODE data sets downloaded from the encode website [53] (https://www.encodeproject.org/). Most of these junctions were shared by another, or by many other, samples within the 70 samples studied for this project. Within this data set 42 samples were collected from normal tissue and 28 samples were from cancerous tissue. Of these tissues 4 were from neuroblastoma, 3 from cervical cancer, 3 from breast cancer, 8 from adenocarcinoma, and 9 from leukemia samples. The final sample of the cancer set was from a liver tumor but was left out of analysis as it lacked any replicates. In order to better classify the splicing events with the most potential for further analysis we further classified splicing events by the difference in the frequency with which they appear in the normal versus cancer samples, allowing for a pseudo-differential expression analysis of these splicing events. For our study we focused on the samples that had over 50% difference occurrence for cancer versus normal samples.

RSF initially aligned the RNA-Seq reads to the hg19 reference genome using the bowtie sequence aligner, version 1.0.1 [54], with the arguments “-p 7 -n 3 -e 112 --un”. These parameters specify the use of 7 threads (−p 7), allow 3 mismatches in the first 28 bases on the high quality end of the read (−n 3), and stipulate that the sum of the Phred quality values across all mismatched positions in the read must not exceed 112 (−e 112). In this step we are interested in reads that do not initially match (−-un).

The RNA-Seq reads in the ENCODE samples we have processed with Read-Split-Fly at this time vary in length from 34 to 101 nt. We selected the minimum split length for each experiment based on the length of the original read (15 nt if <75, 30 nt if ≥75).

These lengths were chosen to balance the resources needed to execute the pipeline with the sensitivity of support for potential splice junction sites.

RSF then aligned these reads with bowtie using parameters “-p 7 --best -m 2 -k 2 -v 0”. We allowed no mismatches (−v 0) and suppressed all alignments if more than 2 alignments are reported (−m 2). With the -v 0 mode, the --best argument instructs bowtie to attempt 800 backtracks instead of the default 125.

RSF next calculated the number of supporting reads at each splice junction site. Potential junction sites are only reported if the splice is between 2 and 50,000 nt long and has at least 2 supporting split reads. For these experiments, we configured RSF to allow for two split reads to support each other if the corresponding left and right ends align within 5 nt of each other. This allows reporting splice junction sites even when there exists reference genome ambiguity due to repeated nucleotide sequences.

### BLAST the U12DB introns and miRNABase miRNAs against the splice junction sequences found by RSF

**Fig. 6:**
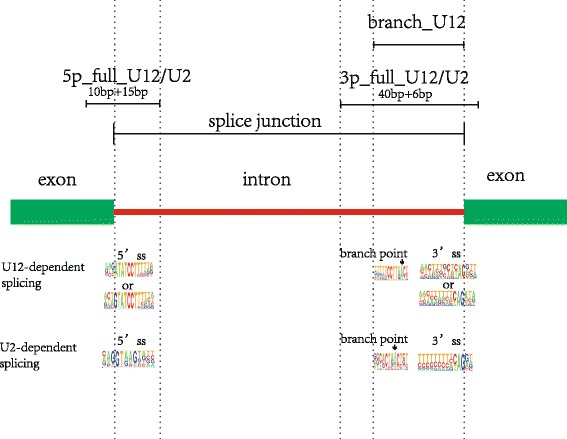
U12/U2 5’splice category. The logos of the figure are adapted from Padgett [61]

The miRNA sequences were downloaded from the miRBase websites [55–60] (http://www.mirbase.org/) Release 21. U12-type and U2-type introns were downloaded from the U12DB website [51] (http://genome.crg.es/cgi-bin/u12db/u12db.cgi), and listed in Additional file 4. The downloaded U12-type and U2-type intron sequences were processed and classified into different categories as queries in the next BLAST step. The customized SQL script was written to retrieve U12-type and U2-type sequences. The categorized query files are delineated in Table 5 and Fig. 6. The logos of the Fig. 6 are adapted from Padgett [61].

**Table 5:** The categorized U12-type and U2-type introns as queries and their stretch of the sequence included

Categorized names	Stretch of sequence included
u12db_3pFull_u12	40 bp of the 3′ acceptor site in the intron and 6 bp of the beginning of the right adjacent exon
u12db_3pFull_u2	40 bp of the 3′ acceptor site in the intron and 6 bp of the beginning of the right adjacent exon
u12db_5pFull_u12	15 bp of the 5′ donor site in the intron and 10 bp of the end of the left adjacent exon
u12db_5pFull_u2	15 bp of the 5′ donor site in the intron and 10 bp of the end of the left adjacent exon
u12db_branch_u12	from 10 bp to the left of the branch site to the 3′ donor site of the intron

A custom C program was written to process the categorized U12-type and U2-type introns and miRNA sequences into FASTA format files. We used BLAST [62] to identify the regions of similarity, using the foregoing categorized U12-type introns, U2-type introns, or miRNA sequences as queries and the spliced sequences discovered by RSF from the 70 human ENCODE samples as subjects of a custom database. RSF ran BLAST by assigning the following parameters - match reward value: 1, mismatch penalty: 1, gap open: 2, gap extend: 2, each expected values: 0.0001, 0.001, 0.01, 0.1, 1, 2, 3, 4, 5, 10, 100, and 1000. The miRNA BLAST reported zero hits for two ENCODE samples, ENCSR000AEK and ENCSR000AET, out of the 70 ENCODE samples. The e-value cutoff of 0.001 is used to select significant miRNA hits from the rest 68 samples.

A custom shell script was written to interpret the result files of the BLAST search. The script tabulates the number of junctions reported by RSF for various splicing categories of U12 and U2-type. The same script is used in downstream analysis for miRBase miRNAs that hit on the splice junction sequences from 70 human ENCODE samples.

### Calculation of sensitivity and specificity

Both specificity and sensitivity are calculated for the junctions found by RSF for each of 70 ENCODE samples. Specificity and sensitivity are calculated for the detected junctions following the metrics: 1) Specificity for RSF detected novel junctions (number of RSF detected novel junctions that are validated by EST/ number of RSF detected novel junctions); 2) Specificity for RSF detected junctions (number of RSF detected junctions that are validated by EST/ number of RSF detected junctions); 3) Sensitivity for RSF detected known junctions (number of RSF detected known junctions/ number of UCSC refFlat file possible junctions of genes present in RSF detected known junctions).

The expression sequence tag (EST) is the standard to determine whether detected junctions, novel and/or known, are supported by experimental validation. The EST data [63] is downloaded from the UCSC table browser (http://genome.ucsc.edu) with the following parameters: clade = Mammal, genome = human, assembly = Feb.2009(GRCh37)/hg19, group = All Tracks, track = human ESTs, table = all_est, region = genome, output format = GTF – gene transfer format [52] A detected junction is considered to be validated by EST if its left boundary is overlapped with at least one EST end within 5 bp size buffer to the left and the right, and its right boundary is overlapped with at least one EST start within 5 bp size buffer to the left and the right. EST was calculated for each ENCODE sample processed by RSF (Fig. 1).

The sensitivity calculations were performed both for all genes with junctions detected by RSF (Fig. 2a), and only taking into account genes with a single isoform listed in the UCSC refFlat file (Fig. 2b). The sensitivity calculations were further broken down based on the Reads Per Kilobase Per Million Mapped Reads (RPKM) of the gene. For each ENCODE sample genes were grouped by those with RPKM lower than 5, those between 5 and 10, those between 10 and 50, those between 50 and 100, and those greater than 100. Sensitivity for all genes in each grouping (bin) were calculated for each ENCODE sample, and as a total for all ENCODE samples processed (Fig. 2c).

## Additional files

Additional file 1: Comparison of running time and memory usage between RSR and RSF for 70 ENCODE samples. This file contains detailed memory and running time comparison for 70 ENCODE samples. (XLSX 18 kb)

Additional file 2: Unique splices compared between tumor and normal data for the ENCODE samples. This file contains unique splice junctions in comparing cancer and normal samples. (XLSX 58 kb)

Additional file 3: Detected miRNA hits on spliced sequences in 70 ENCODE samples. This file contains the total number of hits for each miRNA sequence over spliced sequences from all 70 ENCODE samples. (XLSX 59 kb)

Additional file 4: U12-type and U2-type intron sequences used in the study. This file contains U12-type and U2-type 5′ and 3′ sequences and their associated gene information. (XLSX 69 kb)

## Abbreviations

3p_Full: 3′ splice site
5p_Full: 5′ splice site
AS: Alternative Splicing
BLAST: Basic Local Alignment Search Tool
bp: Base pairs
EST: Expressed Sequence Tag
GB: Gigabyte
GBM: Glioblastoma
GO: Gene Ontology
IGHD: Isolated Growth Hormone Deficiency
miRNA: micro Ribonucleic Acid
MOPD I: Microcephalic Osteodysplastic Primordial Dwarfism Type I
mRNA: messenger Ribonucleic Acid
NMD: None-sense Mediated Decay
NRE: Nuclear Retention Mediation
nt: Nucleotides
QC: Quality Control
RPKM: Reads Per Kilobase Per Million Mapped Reads
RSF: Read-Split-Fly
RSR: Read-Split-Run
RSW: Read-Split-Walk
SL: Stem-loop
snRNA: small nuclear RNA
snRNP: small nuclear ribonucleoprotein
SS: Splice Site
TALS: Taybi-linder Syndrome
UCSC: University of California Santa Cruz

## References

[1] Chen L, Tovar-Corona JM, Urrutia AO (2012). Alternative Splicing: A Potential Source of Functional Innovation in the Eukaryotic Genome. Int J Evol Biol.

[2] Roy B, Haupt LM, Griffiths LR (2013). Review: Alternative Splicing (AS) of Genes As An Approach for Generating Protein Complexity. Curr Genomics.

[3] Yap K, Makeyev EV (2013). Regulation of gene expression in mammalian nervous system through alternative pre-mRNA splicing coupled with RNA quality control mechanisms. Mol Cell Neurosci.

[4] Krebs JE, Goldstein ES, Kilpatrick ST (2013). Lewin's essential genes. Jones & Bartlett Learning titles in biological science.

[5] Benecke H, Luhrmann R, Will CL (2005). The U11/U12 snRNP 65K protein acts as a molecular bridge, binding the U12 snRNA and U11-59K protein. EMBO J.

[6] Burge CB, Padgett RA, Sharp PA (1998). Evolutionary Fates and Origins of U12-Type Introns. Mol Cell.

[7] Levine A, Durbin R (2001). A computational scan for U12-dependent introns in the human genome sequence. Nucleic Acids Res.

[8] Verbeeren J, Niemela EH, Turunen JJ, Will CL, Ravantti JJ, Luhrmann R, Frilander MJ (2010). An ancient mechanism for splicing control: U11 snRNP as an activator of alternative splicing. Mol Cell.

[9] Sheth N, Roca X, Hastings ML, Roeder T, Krainer AR, Sachidanandam R (2006). Comprehensive splice-site analysis using comparative genomics. Nucleic Acids Res.

[10] Patel AA, McCarthy M, Steitz JA (2002). The splicing of U12-type introns can be a rate-limiting step in gene expression. EMBO J.

[11] Niemela EH, Oghabian A, Staals RH, Greco D, Pruijn GJ, Frilander MJ (2014). Global analysis of the nuclear processing of transcripts with unspliced U12-type introns by the exosome. Nucleic Acids Res.

[12] Argente J, Flores R, Gutierrez-Arumi A, Verma B, Martos-Moreno GA, Cusco I, Oghabian A, Chowen JA, Frilander MJ, Perez-Jurado LA (2014). Defective minor spliceosome mRNA processing results in isolated familial growth hormone deficiency. EMBO Mol Med.

[13] Pessa HKJ, Frilander MJ (2011). Minor Splicing, Disrupted. Science.

[14] He L, Hannon GJ (2004). MicroRNAs: Small RNAs with a big role in gene regulation (vol 5, pg 522 2004). Nat Rev Genet.

[15] Mendell JT (2005). MicroRNAs - Critical regulators of development, cellular physiology and malignancy. Cell Cycle.

[16] Krol J, Loedige I, Filipowicz W (2010). The widespread regulation of microRNA biogenesis, function and decay. Nat Rev Genet.

[17] Czech B, Hannon GJ (2011). Small RNA sorting: matchmaking for Argonautes. Nat Rev Genet.

[18] Esteller M (2011). Non-coding RNAs in human disease. Nat Rev Genet.

[19] Carninci P, Kasukawa T, Katayama S, Gough J, Frith MC, Maeda N, Oyama R, Ravasi T, Lenhard B, Wells C (2005). The transcriptional landscape of the mammalian genome. Science.

[20] Esquela-Kerscher A, Slack FJ (2006). Oncomirs - microRNAs with a role in cancer. Nat Rev Cancer.

[21] Hammond S (2007). M: MicroRNA as tumor supressors. Nat Genet.

[22] Croce CM (2009). Causes and consequences of microRNA dysregulation in cancer. Nat Rev Genet.

[23] Nicoloso MS, Spizzo R, Shimizu M, Rossi S, Calin GA (2009). MicroRNAs - the micro steering wheel of tumour metastases. Nat Rev Cancer.

[24] Kim J, Inoue K, Ishii J, Vanti WB, Voronov SV, Murchison E, Hannon G, Abeliovich A (2007). A MicroRNA feedback circuit in midbrain dopamine neurons. Science.

[25] Schaefer A, O'Carroll D, Tan CL, Hillman D, Sugimori M, Llinas R, Greengard P (2007). Cerebellar neurodegeneration in the absence of microRNAs. J Exp Med.

[26] Shin D, Shin JY, McManus MT, Ptacek LJ, Fu YH (2009). Dicer ablation in oligodendrocytes provokes neuronal impairment in mice. Ann Neurol.

[27] Hebert SS, Papadopoulou As Fau - Smith P, Smith P Fau - Galas M-C, Galas Mc Fau - Planel E, Planel E Fau - Silahtaroglu AN, Silahtaroglu An Fau - Sergeant N, Sergeant N Fau - Buee L, Buee L Fau - De Strooper B, De Strooper B: Genetic ablation of Dicer in adult forebrain neurons results in abnormal tau hyperphosphorylation and neurodegeneration. (1460–2083 (Electronic)). 10.1093/hmg/ddq311 20660113

[28] Gehrke S, Imai Y, Sokol N, Lu B (2010). Pathogenic LRRK2 negatively regulates microRNA-mediated translational repression. Nature.

[29] Glinsky GV (2008). An SNP-guided microRNA map of fifteen common human disorders identifies a consensus disease phenocode aiming at principal components of the nuclear import pathway. Cell Cycle.

[30] Wang G, van der Walt JM, Mayhew G, Li YJ, Zuchner S, Scott WK, Martin ER, Vance JM (2008). Variation in the miRNA-433 binding site of FGF20 confers risk for Parkinson disease by overexpression of alpha-synuclein. Am J Hum Genet.

[31] Williams AH, Valdez G, Moresi V, Qi X, McAnally J, Elliott JL, Bassel-Duby R, Sanes JR, Olson EN (2009). MicroRNA-206 delays ALS progression and promotes regeneration of neuromuscular synapses in mice. Science.

[32] Haramati S, Chapnik E, Sztainberg Y, Eilam R, Zwang R, Gershoni N, McGlinn E, Heiser PW, Wills AM, Wirguin I (2010). miRNA malfunction causes spinal motor neuron disease. Proc Natl Acad Sci U S A.

[33] Lee Y, Samaco RC, Gatchel JR, Thaller C, Orr HT, Zoghbi HY (2008). miR-19, miR-101 and miR-130 co-regulate ATXN1 levels to potentially modulate SCA1 pathogenesis. Nat Neurosci.

[34] Chen WL, Lin JW, Huang HJ, Wang SM, Su MT, Lee-Chen GJ, Chen CM, Hsieh-Li HM (2008). SCA8 mRNA expression suggests an antisense regulation of KLHL1 and correlates to SCA8 pathology. Brain Res.

[35] Albinsson S, Suarez Y, Skoura A, Offermanns S, Miano JM, Sessa WC (2010). MicroRNAs are necessary for vascular smooth muscle growth, differentiation, and function. Arterioscler Thromb Vasc Biol.

[36] Zhao Y, Ransom JF, Li A, Vedantham V, von Drehle M, Muth AN, Tsuchihashi T, McManus MT, Schwartz RJ, Srivastava D (2007). Dysregulation of cardiogenesis, cardiac conduction, and cell cycle in mice lacking miRNA-1-2. Cell.

[37] Yang B, Lin H, Xiao J, Lu Y, Luo X, Li B, Zhang Y, Xu C, Bai Y, Wang H (2007). The muscle-specific microRNA miR-1 regulates cardiac arrhythmogenic potential by targeting GJA1 and KCNJ2. Nat Med.

[38] Fang Y, Shi C, Manduchi E, Civelek M, Davies PF (2010). MicroRNA-10a regulation of proinflammatory phenotype in athero-susceptible endothelium in vivo and in vitro. Proc Natl Acad Sci U S A.

[39] Cordes KR, Sheehy NT, White MP, Berry EC, Morton SU, Muth AN, Lee TH, Miano JM, Ivey KN, Srivastava D (2009). miR-145 and miR-143 regulate smooth muscle cell fate and plasticity. Nature.

[40] Ji R, Cheng Y, Yue J, Yang J, Liu X, Chen H, Dean DB, Zhang C (2007). MicroRNA expression signature and antisense-mediated depletion reveal an essential role of MicroRNA in vascular neointimal lesion formation. Circ Res.

[41] Clop A, Marcq F, Takeda H, Pirottin D, Tordoir X, Bibe B, Bouix J, Caiment F, Elsen JM, Eychenne F (2006). A mutation creating a potential illegitimate microRNA target site in the myostatin gene affects muscularity in sheep. Nat Genet.

[42] Sethupathy P, Borel C, Gagnebin M, Grant GR, Deutsch S, Elton TS, Hatzigeorgiou AG, Antonarakis SE (2007). Human microRNA-155 on chromosome 21 differentially interacts with its polymorphic target in the AGTR1 3′ untranslated region: a mechanism for functional single-nucleotide polymorphisms related to phenotypes. Am J Hum Genet.

[43] Gatto S, Della Ragione F Fau - Cimmino A, Cimmino A Fau - Strazzullo M, Strazzullo M Fau - Fabbri M, Fabbri M Fau - Mutarelli M, Mutarelli M Fau - Ferraro L, Ferraro L Fau - Weisz A, Weisz A Fau - D'Esposito M, D'Esposito M Fau - Matarazzo MR, Matarazzo MR: Epigenetic alteration of microRNAs in DNMT3B-mutated patients of ICF syndrome. (1559–2308 (Electronic)). 10.4161/epi.5.5.11999 20448464

[44] Urdinguio RG, Fernandez AF, Lopez-Nieva P, Rossi S, Huertas D, Kulis M, Liu CG, Croce CM, Calin GA, Esteller M (2010). Disrupted microRNA expression caused by Mecp2 loss in a mouse model of Rett syndrome. Epigenetics.

[45] Starczynowski DT, Kuchenbauer F, Argiropoulos B, Sung S, Morin R, Muranyi A, Hirst M, Hogge D, Marra M, Wells RA (2010). Identification of miR-145 and miR-146a as mediators of the 5q- syndrome phenotype. Nat Med.

[46] Wu H, Tao J, Chen PJ, Shahab A, Ge W, Hart RP, Ruan X, Ruan Y, Sun YE (2010). Genome-wide analysis reveals methyl-CpG-binding protein 2-dependent regulation of microRNAs in a mouse model of Rett syndrome. Proc Natl Acad Sci U S A.

[47] Brest P, Lapaquette P, Souidi M, Lebrigand K, Cesaro A, Vouret-Craviari V, Mari B, Barbry P, Mosnier JF, Hebuterne X (2011). A synonymous variant in IRGM alters a binding site for miR-196 and causes deregulation of IRGM-dependent xenophagy in Crohn's disease. Nat Genet.

[48] Lewis MA, Quint E, Glazier AM, Fuchs H, De Angelis MH, Langford C, van Dongen S, Abreu-Goodger C, Piipari M, Redshaw N (2009). An ENU-induced mutation of miR-96 associated with progressive hearing loss in mice. Nat Genet.

[49] Bai Y, Hassler J, Ziyar A, Li P, Wright Z, Menon R, Omenn GS, Cavalcoli JD, Kaufman RJ, Sartor MA (2014). Novel bioinformatics method for identification of genome-wide non-canonical spliced regions using RNA-Seq data. PLoS One.

[50] Bai Y, Kinne J, Donham B, Jian F, Ding L, Hassler J, Kaufman RJ. Read-Split-Run: An improved bioinformatics pipeline for identification of genome-wide non-canonical spliced regions using RNA-Seq data. BMC Genomics. 2016; In Press 10.1186/s12864-016-2896-7 PMC5001233 27556805

[51] Alioto TS (2007). U12DB: a database of orthologous U12-type spliceosomal introns. Nucleic Acids Res.

[52] Kent WJ, Sugnet CW, Furey TS, Roskin KM, Pringle TH, Zahler AM, Haussler D (2002). The human genome browser at UCSC. Genome Res.

[53] Consortium EP (2012). An integrated encyclopedia of DNA elements in the human genome. Nature.

[54] Langmead B, Trapnell C, Pop M, Salzberg SL (2009). Ultrafast and memory-efficient alignment of short DNA sequences to the human genome. Genome Biol.

[55] Griffiths-Jones S. miRBase: microRNA sequences and annotation. Curr Protoc Bioinformatics 2010, Chapter 12:Unit 12 19 11–10. 10.1002/0471250953.bi1209s29 20205188

[56] Griffiths-Jones S (2006). miRBase: the microRNA sequence database. Methods Mol Biol.

[57] Griffiths-Jones S, Grocock RJ, van Dongen S, Bateman A, Enright AJ (2006). miRBase: microRNA sequences, targets and gene nomenclature. Nucleic Acids Res.

[58] Griffiths-Jones S, Saini HK, van Dongen S, Enright AJ (2008). miRBase: tools for microRNA genomics. Nucleic Acids Res.

[59] Kozomara A, Griffiths-Jones S (2014). miRBase: annotating high confidence microRNAs using deep sequencing data. Nucleic Acids Res.

[60] Kozomara A, Griffiths-Jones S (2011). miRBase: integrating microRNA annotation and deep-sequencing data. Nucleic Acids Res.

[61] Padgett RA (2012). New connections between splicing and human disease. Trends Genet.

[62] Healy MD (2007). Using BLAST for performing sequence alignment. Curr Protoc Hum Genet.

[63] Boguski MS, Lowe TM, Tolstoshev CM (1993). dbEST—database for “expressed sequence tags”. Nat Genet.

